# A prospective analysis of pinch grafting of chronic leg ulcers in a series of elderly patients in rural Cameroon

**DOI:** 10.1186/s12895-017-0056-7

**Published:** 2017-03-20

**Authors:** Benjamin Momo Kadia, Christian Akem Dimala, Desmond Aroke, Cyril Jabea Ekabe, Reine Suzanne Mengue Kadia, Alain Chichom Mefire

**Affiliations:** 1 Presbyterian General Hospital Acha-Tugi, Acha-Tugi, Cameroon; 2 Grace Community Health and Development Association, Kumba, Cameroon; 3 Faculty of Epidemiology and Population Health, London School of Hygiene and Tropical medicine, London, UK; 4 Health and Human Development (2HD) Research Group, Douala, Cameroon; 5 Department of Orthopaedics, Southend University Hospital, Essex, UK; 6 Kumba Health District Service, Kumba, Cameroon; 7 Department of Surgery and Obstetrics/Gynaecology, Faculty of Health Sciences, University of Buea, Buea, Cameroon

**Keywords:** Pinch grafting-chronic leg ulcers-elderly

## Abstract

**Background:**

Chronic leg ulcers (CLUs) pose serious public health concerns worldwide. They mainly affect the elderly population. Pinch grafting (PG) could be used to treat a variety of CLUs. However, in Cameroon, there is scarce data on the outcome of PG of CLUs in elderly patients in rural hospitals where most of these patients seek for medical attention and where clinicians rely on unconventional wound dressing methods to treat CLUs. Our objective was to describe the outcome of PG of CLUs in elderly patients in rural Cameroon.

**Methods:**

This was a prospective study conducted in a rural hospital of North West Cameroon. From February 2015 to January 2016, comprehensive historical and clinical data were collected per elderly patient who presented with a chronic leg ulcer necessitating PG. PG was done using a simple procedure and each patient followed up for 8 months. Outcome was described in terms of ulcer healing and pain and donor site complications.

**Results:**

Our series included 13 patients: 8 males (61.54%; 95% CI: 31.58–86.14) and 5 females (38.46%; 95% CI: 13.86–68.42) aged from 69 to 88 years (mean: 77.54 ± 5.70 years). Three patients (23.08%; 95% CI: 5.04–53.81) had associated co-morbidities. All the ulcers were unilateral with durations ranging from 7 to 41 months (mean: 19.46 ± 11.03 months). The ulcers ranged in size from 9.0 to 38.1 cm^2^ (mean: 17.66 ± 8.35 cm ^2^). We registered one (7.69%; 95% CI: 0.19–36.03) graft rejection. Concerning the other ulcers, ten (83.33%; 95% CI: 51.59–97.91) had healed after 12 postoperative weeks while 2 (16.67%; 95% CI: 2.09%–48.41) had healed after 14 postoperative weeks and the mean healing time was 12.33 ± 0.78 weeks. Patients with healed ulcers had reduced ulcer site pain from the immediate postoperative period but there was no significant difference in the mean pain scores before and after graft (6.77 against 4.23, *p =* 0.13). These ulcers remained healed after 8 postoperative months. Each donor site had healed 2 weeks after PG. Donor site problems were minimal and included hypopigmentation.

**Conclusion:**

The outcome of PG of CLUs in our series of older patients was satisfactory. This finding does not discount the role of conservative therapy, but we encourage clinicians in rural Cameroon to consider PG over long-term unconventional conservative therapy in the elderly.

## Background

Chronic leg ulcers (CLUs) pose serious public health concerns worldwide [1–4]. The global proportion of elderly people is on the rise [5] and this segment of the general population is most affected by CLUs [6, 7]. Consequent to the frailty and usual co-morbid states of older people, CLUs in these persons tend to be difficult to manage [8–11]. Prolonged conservative therapy in a bid to definitively treat CLUs is not always efficacious [12, 13], particularly in elderly patients in whom wound healing is usually indolent and/or incomplete [9, 13]. In sub-Saharan Africa, the challenge of managing CLUs in elderly patients is aggravated by the lack of schemes aimed at improving the health-related quality of life of older people who are traditionally left without appropriate care [13]. In view of these, it is imperative to explore and encourage simple, yet effective methods of treating CLUs in elderly people in sub-Saharan Africa.

Previous reports suggest that pinch grafting (PG) could be used to treat a variety of CLUs [14–16]. Some authors have proposed PG as a complement to conservative wound therapy [16] and as first line transplantation technique [17]. It is a simple, safe and cheap procedure which requires minimal resources [12, 15]. Developed nations have even extended the utility of PG to domiciliary basis with remarkable ulcer healing rates [12, 14, 18]. With regards to Cameroon, there is scarce data on the outcome of PG of CLUs in elderly patients in rural areas where most of these patients live and where clinicians still hugely rely on long-term unconventional wound dressing methods to treat CLUs, which usually involve the prolong use of aggressive detergents as well as traditional natural or synthetic bandages, cotton wool and gauzes that keep the wound dry and retard ulcer healing. The objective of our study was to describe the outcome of PG of CLUs in elderly patients in rural Cameroon.

## Methods

This was a prospective study carried out in a Level 1 hospital which is located in a remote village in the North West region of Cameroon. A level 1 hospital is a rural hospital or health center (or a hospital in an extremely disadvantaged urban location) with a small number of beds; it has a sparsely equipped operating room for ‘minor’ procedures; it provides emergency measures in management of 90–95% of trauma and obstetrics cases; and it conducts referral of other patients for further management at a higher level. A chronic leg ulcer was considered as a wound of the leg that persisted for ≥ 3 months [19]. CLUs requiring PG were defined as leg ulcers that showed no tendency to heal after 3 months of conservative therapy using appropriate wound dressing methods. An elderly patient was defined as a person aged 65 years or above [20, 21]. From January 2015 to January 2016, elderly patients with CLUs necessitating PG were studied. Each patient was assessed by the same 3 General practitioners working in the hospital. Patients who died before the end of the pre-specified postoperative follow-up period were excluded. CLUs were described in terms of 2 broad aspects:

- the ulcer itself: onset, duration, position, multiplicity, tenderness, temperature, size (length X width X π/4) [22], edge, base, depth, and discharge (if present);
- surrounding tissues: state of adjacent tissue, local circulation and innervation

Ulcer pain was graded using a validated 10 echelon visual analogue scale (VAS).

Ethics approval for this study was obtained from the Institutional Review Board of the Faculty of Health Sciences, University of Buea, Cameroon. All the participants signed an informed consent form prior to data collection for the study.

### Pre-graft procedure

Conservative therapy (mainly by ulcer debridement and dressing) was done until enough granulation tissue was generated on the ulcer surfaces. Antibiotics were not systematically used but in 2 of the 13 patients, antibiotics were administered for cellulitis.

### Graft procedure

**Fig. 1:**
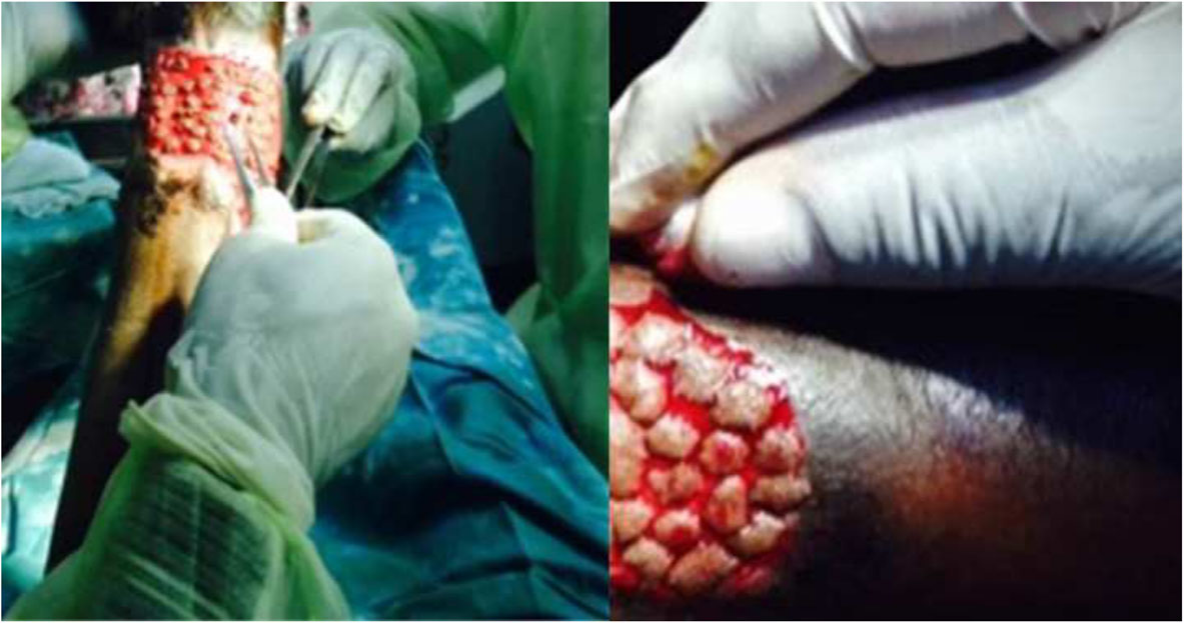
Grafting of an ulcer

PG in our series was done by the same (2) general practitioners. The anterior aspect of the ipsilateral thigh was used in all cases as the donor site. The site was prepared with Cetrimide solution and Povidone iodine and an area of skin roughly equivalent to the ulcer was demarcated for obtainment of the grafts. The demarcated zone was locally anaesthetized by superficial infiltration with 2% Lidocaine combined with epinephrine. Using a syringe needle inclined to about 30 degrees, tents of skin were raised and cut using a scalpel to harvest multiple small split-thickness grafts whose depths were limited to the dermal layer. These varied from 15 to 35 pinches in our series. The grafts were then put inside a petri dish containing 0.9% saline. Lidocaine combined with epinephrine was applied on the surface of the donor site to achieve faster haemostasis. Vaseline gauze imbibed in 0.9% saline and compressive dressings were then applied on the donor site. The dermal surfaces of the grafts were placed on the ulcer a few mm apart (Fig. 1) and covered with two taut vaseline gauze sheets imbibed with 0.9% saline. Two layers of mild compressive dressing were then applied.

### Post-graft procedure

Postoperatively, the patients remained relatively still in bed for one week to avoid shearing forces on the grafts. The donor site was left untouched for a week while the recipient site was uncovered after 5 days and moistened daily with Vaseline gauze imbibed in 0.9% saline. Patients were discharged home after one week of continuous moist dressings of the recipient site, and individually reviewed on outpatient basis: on a weekly basis for the first month, after every two weeks for the second month and then monthly thereafter. Each patient was assessed over 8 postoperative months. Outcome was described in terms of ulcer healing and pain and donor site complications.

### Statistical analysis

The data collected was analyzed using Epi Info version 7 statistical software and means, 95% confidence intervals, proportions and standard deviations were recorded. Means and percentages before and after grafting were compared using the student-t-test.

## Results

One patient died at the sixth postoperative month and was excluded from the study. Table 1 summarizes the preoperative characteristics of the remaining 13 patients (92.86% inclusion rate). Our series included 8 males (61.54%; 95% CI: 31.58-86.14) and 5 females (38.46%; 95% CI: 13.86-68.42) aged from 69 to 88 years (mean: 77.54 ± 5.70 years). Three patients (23.08%; 95% CI: 5.04-53.81) had associated co-morbidities. The durations of the ulcers (including the period of conservative therapy) ranged from 7 to 41 months (mean: 19.46 ± 11.03 months). The ulcers ranged in size from 9.0 to 38.1 cm^2^ (mean: 17.66 ± 8.35 cm ^2^). Local circulation in tissues surrounding the ulcers was intact in all the patients except in case 8. Local innervation was conserved in all the affected limbs: neurological examination revealed that muscle tone and power as well as sensation to touch, pain, and vibrations were normal in all the affected limbs.

**Table 1:** Characteristics of patients

Patient S/N			Variable
	Gender/age	Social habits/comorbidity	Ulcer
1	M/77y		18 month-old superficial traumatic ulcer on medial aspect of middle third of leg with purulent discharge. Ulcer was 13 cm2 in size with an infected soft tissue base. It was severely tender. Adjacent tissue was normal.
2	M/78y		13 month-old deep spontaneous ulcer above medial malleolus. Ulcer had smooth regular sloppy edges, a subcutaneous tissue base and covered an area of 14 cm2. It was warm and moderately tender. Surrounding tissue was normal. Probable aetiology: ischaemia
3	F/75y	• Hypertension	14 month-old superficial traumatic ulcer on medial aspect of middle third of leg. Ulcer had smooth irregular flat edges and covered an area of 21 cm2. It was severely tender and its base consisted of subcutaneous tissue. Surrounding tissue was normal.
4	M/86y		12 month-old superficial traumatic ulcer just above medial malleolus with purulent discharge. Ulcer was 9.0 cm2 in size, mildly tender and had infected soft tissue base with maggots. Surrounding tissue was normal.
5	F/76y		9 month-old deep traumatic ulcer on anterior aspect of middle third of leg with purulent discharge. It covered an area of 23 cm2 with an infected subcutaneous tissue base and was severely tender. Surrounding tissue up to knee level was erythematous, oedematous and severely tender (suggestive of cellulitis)
6	F/77y		25 month-old superficial traumatic ulcer on anterior aspect of middle third of leg. It covered an area of 15 cm2 with a subcutaneous tissue base. It was severely tender. Surrounding tissue was normal.
7	F/70y		41 month-old deep spontaneous ulcer just above lateral malleolus. It covered an area of 20 cm2 with a subcutaneous tissue base. It was severely tender. Surrounding tissue was normal.
8	M/73y		30 month-old superficial traumatic ulcer involving posterior third of distal leg. It was 27 cm2 in size and its base consisted of subcutaneous tissue. It was severely tender. Surrounding tissue was normal.
9	M/81y		39 month-old deep spontaneous ulcer involving almost the entire circumference of the distal third of the leg. It was 38.1 cm2 in size with smooth sloppy (regular) edges and a subcutaneous tissue base. It was cold and severely tender. Surrounding tissue was normal but for faint dorsalis pedis and posterior tibial pulses. Probable aetiology: ischaemia
10	M/83y		18 month-old superficial traumatic ulcer above medial malleolus. Ulcer was 10.0 cm2 in size with a subcutaneous tissue base. It was moderately tender. Surrounding tissue was normal.
11	M/88y		15 month-old superficial spontaneous ulcer on dorsum of foot with serous discharge. Ulcer was 9.3 cm2 in size and moderately tender. Its base was comprised of subcutaneous tissue. Surrounding tissue up to mid leg was erythematous, oedematous and severely tender (suggesting cellulitis). Probable aetiology: ischaemia.
12	M/75y	• Chronic smoking • Diabetes	7 month-old superficial traumatic ulcer just above lateral malleolus with purulent discharge. Ulcer was 11.4 cm2 in size and severely tender. Its base consisted of infected subcutaneous tissue and surrounding tissue was normal.
13	F/69y	• Hypertension	12 month-old superficial traumatic ulcer on posterolateral aspect of distal third of leg. Ulcer measured 18.8 cm2 in size. Its base comprised of subcutaneous tissue. Ulcer was moderately tender. Surrounding tissue was normal.

*S/N* Serial number, *y* yearsm, *M* male, *F* female

The pregraft ulcer pain scores ranged from 4 to 9 (mean: 6.77) with 92.31% (95% CI: 63.97%-99.81%) having ulcer pain scores ≥ 5. Nine of the 13 ulcers were traumatic while 4 were probably ischaemic (and ischaemic) ulcers. Nine of the ulcers were due to trauma, and 4 probably due to ischaemia.

**Fig. 2:**
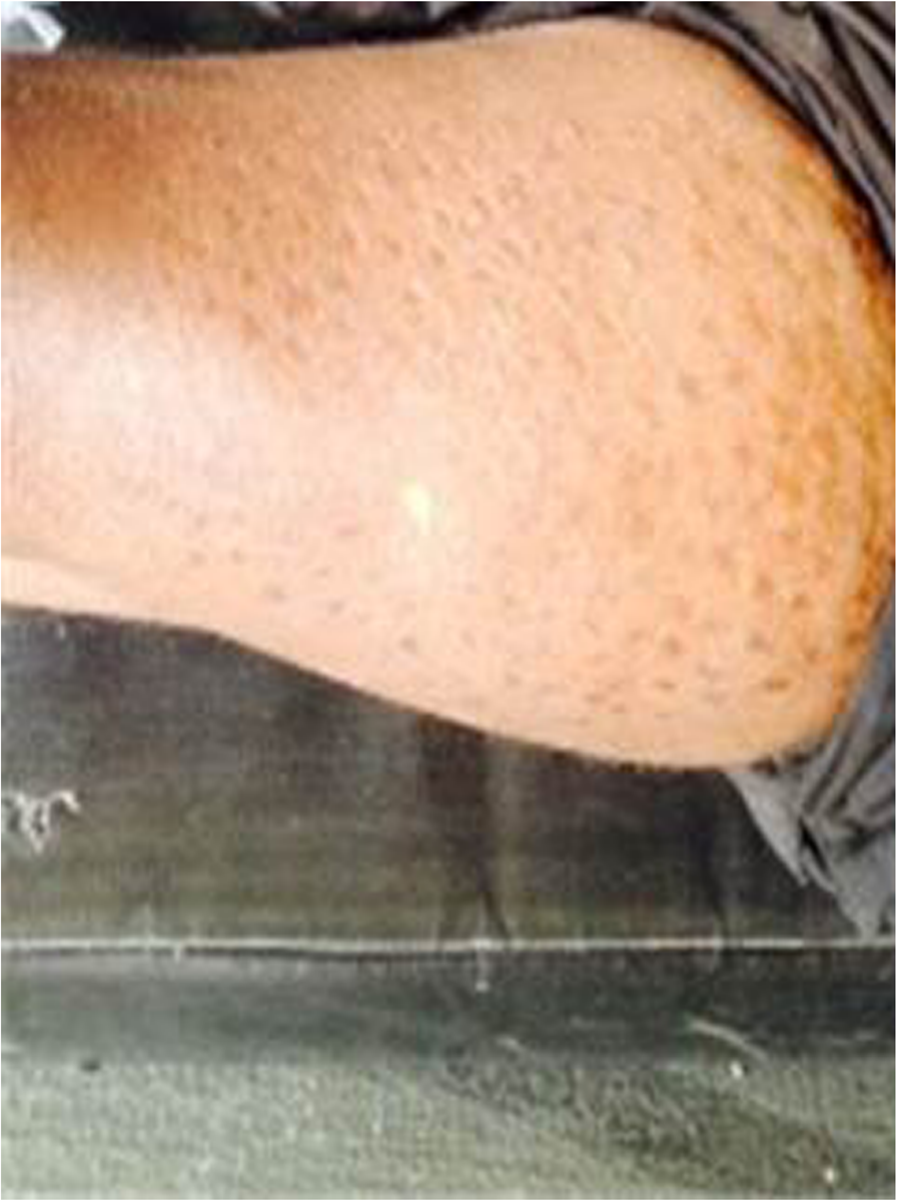
Hypo-pigmented donor site

In the 12^th^ case, there was partial graft rejection by the fifth postoperative day which progressed to complete graft rejection by the second postoperative week. Concerning the other ulcers, ten (83.33%; 95% CI: 51.59-97.91) had healed after 12 postoperative weeks while 2 (16.67%; 95% CI: 2.09%-48.41) had healed after 14 postoperative weeks. The mean healing time was 12.33 weeks ± 0.78 weeks. The healed ulcers remained healed throughout the follow up period. Post graft, the pain scores ranged from 2-9 (mean: 4.23) and although only 7.69% (95% CI: 0.19–36.03%) of the patients had pain scores ≥ 5, this was not significantly different from the 92.31% of patients with pre-graft pain scores ≥ 5 (*p =* 0.92 from Fischer’s exact test). There was no significant difference in the mean pain scores before and after graft (6.77 against 4.23, *p =* 0.13). None of the patients developed new ulcers during the follow-up period. Donor sites were all healed by the end of the second postoperative week. Donor site problems remained minimal and included mild hypopigmentation (Fig. 2).

## Discussion

While re-iterating the important role of PG in the treatment of CLUs in the elderly, the current report, to the best of our knowledge, is the first to assess the technical aspects and long term outcome of PG performed on a series of relatively older patients managed using simple resources at a rural hospital facility of Cameroon. Considering the lower age cut-off for defining older people in the African context [21] and the mean age of our patients, our cohort could be regarded as very elderly. The average healing rate of the ulcers was satisfactory and donor site problems were minimal. The low prevalence of comorbidities could explain in part why such a cohort had a satisfactory outcome. It is, however, worth noting that due to lack of more appropriate measures, our assessment of ulcers (for instance, in terms of neurovascular integrity of surrounding tissues) preoperatively was, in part, subjective and comorbidities may have been underdiagnosed in our series.

Initial conservative therapy is indispensable in achieving sufficient wound granulation which is an important pre-requisite for a skin graft to be supported [23]. A limitation common to most small hospitals like ours is the inability to perform cultures [22] from ulcer swabs which is vital in ruling out ulcer base infection, particularly with group A β-haemolytic streptococci. These microbes are notorious for causing graft failure [23]. The complete graft rejection we encountered was possibly due to residual infection at the ulcer base.

Establishing the aetiology of a chronic ulcer is crucial in individualizing and optimizing health care especially in older patients who usually have underlying comorbidities [2, 4]. Importantly, every chronic ulcer unresponsive to conservative therapy should be biopsied in order to rule out malignant changes [23]. However, even in the presence of robust diagnostic facilities, the aetiologies of most CLUs tend to be unknown [8] or multifactorial [9, 24] and from a practical perspective, verifying the exact aetiology of every chronic ulcer does not always seem to take precedence over performing surgery especially in the event of failing conservative treatment and/or intractable pain. CLUs in our series were predominantly traumatic, which is unusual although consistent with preliminary African reports on relatively younger and more active persons [3]. Traumatic ulcers tend to become chronic in poor settings because of initial unskilled management [13]. The limited size of our cohort prevents us from drawing valid interpretations with regards to the location of CLUs on the right leg in all our patients. Nevertheless, contemporary studies with larger cohorts (although relatively younger patients) did not find CLUs to have a predilection for a particular leg [3, 13].

The surgical approach we utilized is in line with what was proposed by J.S. Davis in 1930[18]. Although a simple procedure that has been used over several years [14], PG in recent times necessitates extreme caution with regards to donor sites because donor-site morbidity post-graft is of growing concern worldwide [10, 25]. We recorded minimal donor site problems in our series possibly because of the moistening effects of Vaseline gauze (imbibed in saline). Literature inclines towards the use of moist dressings for donor and recipient sites because of faster healing when compared to dry dressings [26].

Sufficient new vascularization of the ulcer bed is expected to have occurred by the third to fifth post-operative day [23]. Thus, in our series, recipient sites were partially uncovered by the fifth postoperative day given that grafts will only take on ulcer beds on which they can become vascularized. However, at this stage, moist dressings must be applied with caution because the epidermal-dermal interface of epithelializing wounds is weak and even minimal shear forces could lead to graft failure [23].

Assessing ulcer site pain in our cohort was deemed very necessary because it is reported that ulcer pain seems to receive less attention in ulcer management and thus individual needs might not be adequately addressed [8]. In order to have an appraisal of the ulcer site pain intensity before and after PG, we used a validated VAS with 10 echelons which was simple enough for our elderly patients to comprehend. We observed a rapid general drop in ulcer site pain after grafting which is a reported merit of PG [14].

**Fig. 3:**
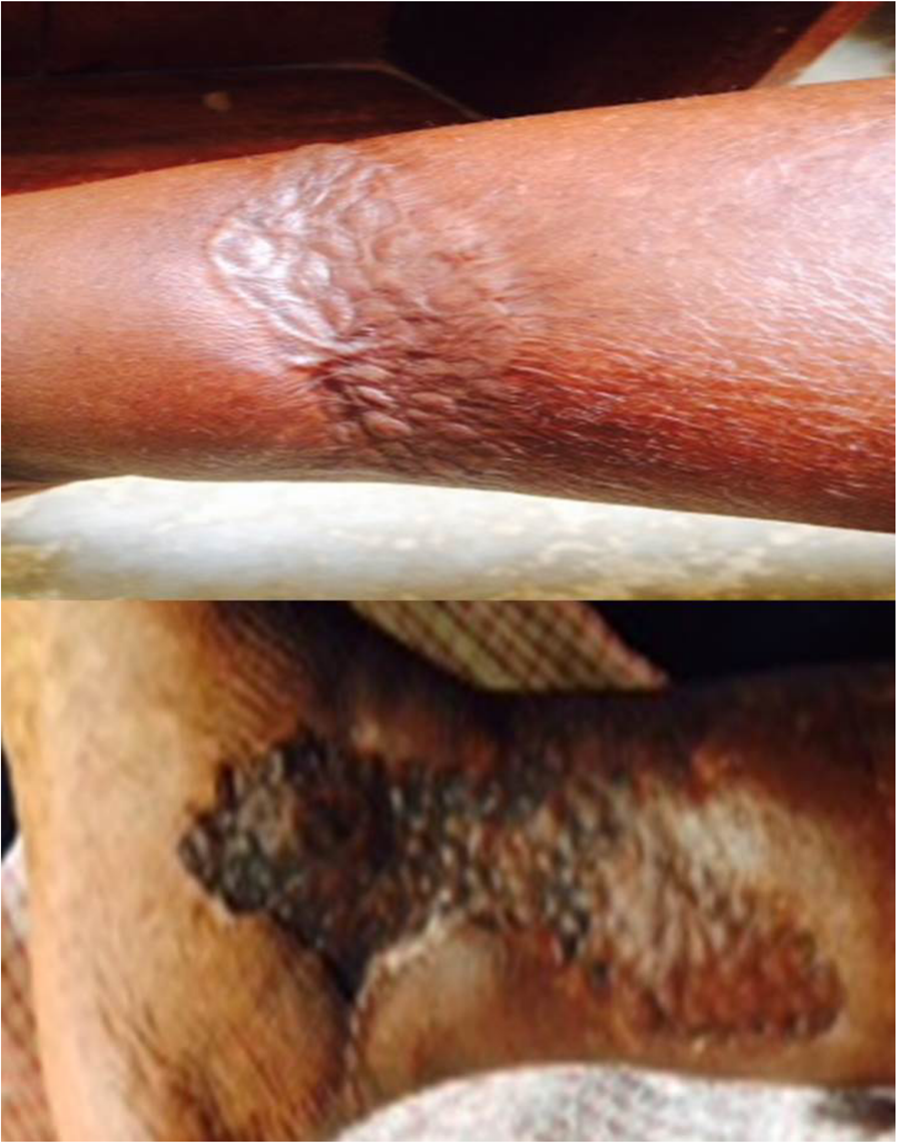
Healed ulcers post-graft

Well known demerits of PG are altered skin pigmentation and graft contraction (Fig. 3). These are sequelae that are inherent to PG and they are consequent to the thin nature of the harvested grafts [27]. Our choice of donor site as the thigh was advantageous in that the discolouration of the donor site due to healing was subsequently covered by hair growth. Females, however, do not benefit from this advantage. The poor cosmesis of the grafted sites following PG remains a subject of concern. That notwithstanding, the recommended attitude is to concurrently consider aesthetic issues and improvement in life quality as well as functionality in order to have a full appraisal of the therapeutic consequences of any skin graft [28, 29].

There is much controversy with regards to where and how to manage CLUs [3]. Our experience, however, suggests that management of CLUs in very elderly people could be adapted context-wise. The population segment most affected by CLUs (elderly people) is generally inactive and home-ridden. A possible prospect therefore is to assess the role of PG on elderly people at a community level in order to make clearer inferences.

## Conclusions

We describe, to the best of our knowledge, the first report assessing PG of CLUs in older patients in rural Cameroon. PG in our series of relatively older patients (who had a low rate of co-morbidities) in a low-income setting was technically feasible and the outcome was satisfactory. These findings do not discount the role of conservative therapy. However, prolonged unconventional conservative management as definitive treatment for all CLUs in elderly patients may not be worthwhile. More reports on the experience of clinicians on the subject matter could possibly create a platform for progressive refinements in PG procedures and help address the dismal burden of CLUs in elderly patients in rural Cameroon.

## Abbreviations

CI: Confidence Interval
CLUs: Chronic leg ulcers
F: Female
M: Male
PG: Pinch Grafting
S/N: Serial number
VAS: Visual Analogue Scale
y: Years

## References

[1] Adeyemi A, Muzerengi S, Gupta I (2009). Leg ulcers in older people: a review of management. Br J Med Pract.

[2] Rayner R, Carville K, Keaton J, Prentice J, Santamaria N (2009). Leg ulcers: atypical presentations and associated comorbidities. Wound Pract Res.

[3] Rahman GA, Adigun IA, Fadeyi A (2010). Epidemiology, etiology, and treatment of chronic leg ulcer: Experience with sixty patients. Ann Afr Med.

[4] Tricco AC, Cogo E, Isaranuwatchai W, Khan PA, Sanmugalingham G, Antony J, Hoch JS, Straus SE. A systematic review of cost-effectiveness analyses of complex wound interventions reveals optimal treatments for specific wound types. BMC Med. 2015;13:90. 10.1186/s12916-015-0326-3 PMC4405871 25899057

[5] Retamar P, Lopez-Prieto MD, Rodriguez-Lopez F, de Cueto M, Garcia MV, Gonzalez-Galan V (2014). Predictors of early mortality in very elderly patients with bacteraemia: a prospective multicenter cohort. Int J Infect Dis.

[6] Briggs M, Jose CS (2003). The prevalence of leg ulceration: a review of the literature. EWMA J.

[7] Adam D, Naik J, Hartshorne T, Bello M, M L. Diagnosis and management of 689 chronic leg ulcers in a single-visit assessment clinic. Eur J Vasc Endovasc Surg. 2003;25:462–8. 10.1053/ejvs.2002.1906 12713787

[8] Hellström A, Nilsson C, Nilsson A, Fagerström C. Leg ulcers in older people: a national study addressing variation in diagnosis, pain and sleep disturbance. BMC Geriatrics. 2016;16:25. 10.1186/s12877-016-0198-1 PMC4722676 26797291

[9] Gohel M, Taylor M, Earnshaw J, Heather B, Poskitt K, Whyman M (2005). Risk factors for delayed healing and recurrence of chronic venous Leg ulcers-an analysis of 1324 legs. Eur J Vasc Endovasc Surg Elsevier.

[10] Rogers AD, Atherstone AK, Rode H (2009). Over grafting donor site. East Cent African J Surg.

[11] Ho L, Bailey B, Bajaj P (1976). Pinch grafts in the treatment of chronic venous Leg ulcers. Chir Plast.

[12] Steele K (1985). Pinch grafting for chronic venous leg ulcers in general practice. J R Coll Gen Pract.

[13] Adigun IA, Rahman GA, Fadeyi A (2010). Chronic Leg ulcer in the older Age group: etiology and management. Res J Med Sci Medwell J.

[14] Oien RF, Hansen BU, Hakansson A (1998). Pinch grafting of leg ulcers in primary care. Acta Derm Venereol Scandinavian Univ Press.

[15] Jayaseelan E, Aithal VV (2004). Pinch skin grafting in Non-healing leprous ulcers. Int J Lepr.

[16] Christiansen J, Lorens E, Tegner E (1997). Pinch grafting of Leg ulcers: a retrospective study of 412 treated ulcers in 146 patients. Acta Derm Venereol.

[17] Ahnlide I, Bjellerup M (1997). Efficacy of pinch grafting in Leg ulcers of different aetiologies. Acta Derm Ve.

[18] Davis J (1930). The small deep graft. Ann Surg Elsevier.

[19] Agale SV. Chronic Leg Ulcers: Epidemiology, Aetiopathogenesis, and Management. Ulcers. 2013;2013:e413604.

[20] Sieber CC. [The elderly patient--who is that?]. Internist (Berl). 2007;48(11):1190, 1192–4. 10.1007/s00108-007-1945-3 17934704

[21] World Health Organization (2016). Health statistics and information system.

[22] JArroll B, Bourchier R, Gelber P, Jull A, Latta A, Milne R, Oliver F, Tuuta N, Walker N, Waters J. Care of people with chronic leg ulcers: An evidence based guideline. New Zealand: New Zealand Guidelines Group; 1999.

[23] Goodacre T. Plastic and reconstructive surgery. In: Norman W, Christopher B, O’connel P (eds.) Bailey and Love’s SHORT Practice of Surgery. 25th ed. London. Edward Arnold Ltd; 2008. p394–4091

[24] Oien R, Hakansson A, Hansen B (2001). Leg ulcers in patients with rheumatoid arthritis-a prospective study of aetiology, wound healing and pain reduction after pinch grafting. Rheumatology.

[25] Otene C, Olaitan P, Ogbonnaya I, Nnabuko R (2011). Donor site morbidity following harvest of split-thickness grafts in South Eastern Nigeria. J West African Coll Surg.

[26] Wiechula R (2003). The use of moist wound-healing dressings in the management of split-thickness skin graft donor sites: a systematic review. Int J Nurs Pract.

[27] Shimizu R, Kishi K. Skin Graft. Plastic Surgery International. 2012;2012:e563493. 10.1155/2012/563493 PMC3335647 22570780

[28] Martin P, S L. Inflammatory cells during wound repair: the good, the bad and the ugly. Trend Cell Biol. 2005;15:599–607. 10.1016/j.tcb.2005.09.002 16202600

[29] Metcalfe A, Ferguson M (2007). Bioengineering skin using mechanisms of regeneration and repair. Biomaterials.

